# The COVID-19 Mirage: A Young Biologist With an Atypical Presentation of a Zoonotic Disease During the COVID-19 Pandemic

**DOI:** 10.7759/cureus.26493

**Published:** 2022-07-01

**Authors:** Jose D Chiriboga, Jonathan Garcia, Daniel Garcia, Santiago Mena, Jose E Leon-Rojas

**Affiliations:** 1 “Incubadora de Investigación en Medicina” (InMed), NeurALL Nest, Quito, ECU; 2 Maestria en Investigación en Ciencias de la Salud, Universidad Internacional del Ecuador, Quito, ECU; 3 School of Medicine, Faculty of Health and Life Sciences, Universidad Internacional del Ecuador, Quito, ECU

**Keywords:** atypical, delusion, zoonotic, leptospirosis, covid-19

## Abstract

COVID-19 has become one of the main causes of febrile illness among emergency department patients and is always a differential diagnosis to keep in mind. Nonetheless, some patients with a history of exposure, persistent fever, and suspicion of COVID-19 end up having entirely different etiologies. Here, we present the case of a 29-year-old male biologist with an uncommon presentation of a zoonotic disease, characterized by unspecific signs and symptoms, which led to a delayed diagnosis, causing significant emotional distress in the patient. We also coin the term “COVID-19 Mirage," to serve as a constant reminder for clinicians of the effect that COVID-19 has caused on the differential diagnosis of fever of unknown etiology.

## Introduction

The COVID-19 pandemic has affected 531,588,094 people throughout the world and has caused a total of 6,297,346 deaths as of June 02, 2022 [1]. In Ecuador, 878,196 persons have been infected of whom 35,645 have died [2]. Certainly, the numbers showcase the tremendous impact of severe acute respiratory syndrome coronavirus 2 (SARS-CoV-2) throughout the world, which is still being considered by clinicians in every country as an important differential diagnosis when evaluating a patient with fever. Amidst the ongoing SARS-CoV-2 pandemic, the management of cases of fever of unknown origin and nonspecific signs and symptoms of infectious diseases has been a real challenge for healthcare professionals; patients who are not infected with SARS-CoV-2 tend to have delayed diagnoses of their respective infectious disease, which increases the economical and psychological burden for the affected patient [3].

However, fever alone, without any associated signs or symptoms, will not immediately prompt suspicion of COVID-19 in the clinician’s mind; but what if fever is the sole symptom in a patient who had close contact with a COVID-19-positive individual? Moreover, what if the patient has multiple negative COVID-19 rapid tests? Is COVID-19 still on the table?

Here, we present the case of a 29-year-old male biologist with an atypical presentation of a zoonotic disease, a close contact with a COVID-19 positive case (talking without a mask at less than one meter and sharing a water bottle), and repeatedly testing negative for COVID-19 by rapid antigen test in Ecuador, South America. We also coin the term “COVID-19 Mirage” as a way to remind clinicians that not everything that burns (fever), even during the pandemic, is COVID-19 and that patients need to be carefully evaluated before isolation to prevent the spread of SARS-CoV-2.

## Case presentation

A 29-year-old male biologist, without any previous medical illness other than a past COVID-19 infection, presented with a 24-hour history of malaise, fatigue, and low-grade fever (37.8 ºC); he also had a history of close contact (talking without a mask at less than one meter and sharing a beverage) with a PCR (polymerase chain reaction)-positive COVID-19 case five days prior to the beginning of his symptoms. The patient underwent an antigen test that yielded a negative result. He had traveled to the Ecuadorian jungle three to four weeks prior to collect samples in order to test the water quality of rivers in the region as part of his job as a freelance environmental consultant. He denied exposure to wildlife (other than fish and river fauna, and mosquito bites); he also denied any spider or snake bites. He reported a similar clinical presentation in the past when he got infected with SARS-CoV-2 at the beginning of the COVID-19 epidemic in Ecuador.

Physical examination revealed normal vital signs (BP: 115/75 mmHg, HR: 75 bpm, RR: 11 bpm, SpO_2_: 95%) with a temperature of 38ºC. There was no skin or mucous discoloration, no skin rash or lesions, no hepatomegaly or splenomegaly, and no lymphadenopathy; the cardiac and lung auscultation was normal. The only relevant aspect of the physical exam was a mild discomfort upon palpation of the right upper abdominal quadrant (right hypochondrium). A COVID-19 antigen test was repeated, by the patient, 48 hours after symptom onset and again yielded a negative result. A PCR test for COVID-19 and influenza A/B was ordered 72 hours after symptom onset, which confirmed the negative results shown by the two prior antigen tests and ruled out a seasonal respiratory virus as the cause of fever. Furthermore, three sets of laboratory tests were performed in order to detect the source of fever (Table 1 shows the results of the tests), with the main differential diagnosis being a tropical disease contracted due to his profession as a biologist and direct contact with bodies of water (sampling of water and river fauna).

**Table 1:** Serial laboratory results in our patient HIV: Human immunodeficiency virus; HBsAg: Hepatitis B surface antigen; HB: Hepatitis B; HCV: Hepatitis C virus; N/A: Not applicable, the test was not performed at that sampling moment. The first sample, second sample, and third sample occurred at 3, 4, and 10 days after symptom onset, respectively.

Laboratory Test	First Sample	Second Sample	Third Sample	Reference Values
White blood cells	6150.00/mm^3^	6800.00/mm^3^	N/A	4400.00-11500.00
Neutrophils	4735.50/mm^3^	5596.40/mm^3^	N/A	2000.00-8000.00
Lymphocytes	781.05/mm^3^	639.20/mm^3^	N/A	1000.00-4400.00
Monocytes	541.20/mm^3^	448.80/mm^3^	N/A	80.00-880.00
Basophils	18.45/mm^3^	20.40/mm^3^	N/A	0.00-110.00
Eosinophils	43.05/mm^3^	88.40/mm^3^	N/A	80.00-440.00
Immature granulocytes	0.031 x 10^3^/mm^3^	0.007 x 10^3^/mm^3^	N/A	0.00-0.070
Red blood cells	4800.00 x 10^3^/mm^3^	4780.00 x 10^3^/mm^3^	N/A	4500.00-6400.00
Hemoglobin	15.1 g/dL	15.2 g/dL	N/A	13.6-17.5
Hematocrit	43.4%	43.0%	N/A	40.0-52.0
Mean corpuscular volume	90.4 fL	90.0 fL	N/A	76.00-96.00
Platelets	154.00 x 10^3^/mm^3^	153.00 x 10^3^/mm^3^	N/A	150.00-450.00
Alanine transaminase (ALT)	N/A	92.90 U/L	N/A	15.00-59.00
Aspartate transaminase (AST)	N/A	72.50 U/L	N/A	9.00-50.00
Total bilirubin	N/A	1.94 mg/dL	N/A	0.20-1.30
Direct bilirubin	N/A	1.11 mg/dL	N/A	0.00-0.40
Indirect bilirubin	N/A	0.83 mg/dL	N/A	-
Gamma-glutamyl transferase (GGT)	N/A	315.00 U/L	N/A	8.0-70.0
Alkaline phosphatase (ALP)	N/A	338.0 U/L	N/A	38.0-126.0
Prothrombin time	N/A	11.2 segs	N/A	9.9-11.8
Partial thromboplastin time	N/A	34.4 segs	N/A	23.4-36.2
Ferritin	N/A	938.30 ng/mL	N/A	30.00-400.00
C-reactive protein	N/A	178.50 mg/L	N/A	0.00-10.00
Creatinine	1.01 mg/dL	N/A	N/A	0.60-1.30
Urinalysis	Negative for infection, RBCs, or crystals		N/A	-
HIV 1 & 2	N/A	0.24	N/A	Negative < 0.9
Anti-HAV IgM	N/A	0.01	N/A	Negative < 0.4
HBsAg	N/A	0.59	N/A	Negative < 1.0
Anti-HB Core IgM	N/A	Negative	N/A	
Anti-HCV	N/A	0.05	N/A	Negative < 1.0
Malaria and blood parasites (blood smear)	N/A	Negative	N/A	-
Dengue IgM	N/A	Negative	N/A	-
Leptospiral IgG and IgM	N/A	Negative	Positive IgM	-
Stool analysis	Blastocystis hominis		N/A	-
Blood culture	N/A	Pending	Negative	-

The first sample revealed lymphopenia without leukocytosis or anemia; the renal function was normal. By the time of the reception of the first results, the patient started complaining of increasing pain in the right hypochondrium and bloating after fatty meals; physical examination revealed increased tenderness under the right ribcage but no hepatomegaly. The second set of exams focused on evaluating liver function and injury was requested and showed hyperbilirubinemia with elevated direct bilirubin, increased alanine transaminase (ALT), aspartate transaminase (AST), gamma-glutamyl transferase (GGT), and alkaline phosphatase (ALP), with normal coagulation times pointing toward the possibility of cholestasis. Furthermore, plausible tropical and common infectious diseases were assessed in this sample, and all came back negative (Hepatitis A, B, and C, malaria, blood parasites, dengue, leptospirosis, and HIV).

**Figure 1:**
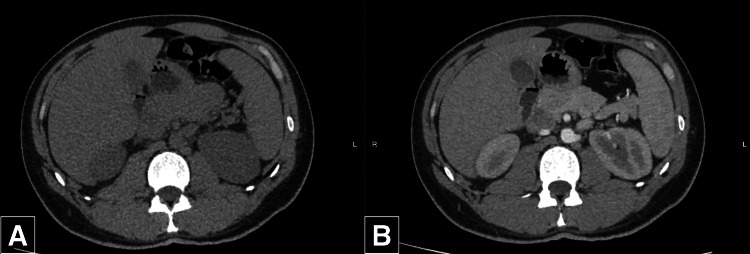
Abdominal CT scan for evaluation of potential liver abscess Panels A and B show non-contrast and contrast-enhanced abdominal CT scans, respectively. No signs of lesion of the hepatic parenchyma can be seen, and all other abdominal structures are normal.

Due to the cholestatic and liver injury findings of the laboratory exams, liver abscess or cholangitis was suspected, and an abdominal contrasted computed tomography (CT) scan was performed five days after symptom onset (Figure 1). An MRI scan was not requested due to the limited availability. The scan showed a liver of normal size and morphology without any lesions within the hepatic parenchyma and a distended gallbladder with thin walls and surrounding edema; the rest of the scan was normal. At this time, the patient persisted with fever (38.5ºC) despite the administration of dual antipyretic oral therapy (Ibuprofen 600 mg three times a day [T.I.D.] and Acetaminophen 1000 mg T.I.D.), prompting his hospitalization.

At the hospital, an abdominal ultrasound was requested and revealed a normal gallbladder without any signs of edema or obstruction; bile ducts were not dilated, and there were no foci of hypervascularity on the doppler scan; additionally, the blood culture came back negative. Therefore, empiric broad-spectrum intravenous (IV) antibiotic therapy with ceftriaxone 2000 mg once a day (Q.D.) and metronidazole 500 mg T.I.D. was prescribed. The patient remained for observation and showed significant clinical improvement. Ten days after symptom onset, while hospitalized, the leptospiral antibody test was repeated and came back positive; the patient’s antibiotic treatment was changed to only IV ceftriaxone 2000 mg Q.D. until completing seven days of treatment at which time the patient was discharged without any symptoms. One week after hospitalization, the patient remained without fever or the need for oral medication. The only complaint was mild abdominal discomfort after eating fatty meals.

## Discussion

Here, we presented the case of a young patient with an uncommon presentation of a zoonotic disease in the context of the COVID-19 pandemic, which led to a delayed diagnosis and treatment. The objective of our case is to showcase the significant effect that SARS-CoV-2 has caused in the diagnosis and treatment of infectious diseases. Certainly, during the pandemic, suspicion of SARS-CoV-2 infection is always on the clinician’s mind, even more, when the patient presents with fever and a history of positive COVID-19 contact. However, we believe that clinicians are experiencing what we have termed the “COVID-19 Mirage.” A mirage is something that appears to be real or possible but is not in fact real. In our case, we assumed that the patient had COVID-19 despite two negative rapid-test results due to his history of a positive contact, the lack of specificity of his symptoms, and the unreliability of tests in our country, which led to a delay in his diagnosis. We propose that “COVID-19 Mirage” appears when the clinical presentation is unspecific or uncommon; this leads the clinician to readily suspect COVID-19 while possibly ignoring other infectious etiologies. We wanted to coin this term and present a case where it happened in order to educate clinicians that “not everything that burns (fever) is COVID-19.”

In 1886, Adolf Weil described a zoonosis later identified as leptospirosis [4]. *Leptospira *is a motile spirochete with hair-like structures with hooked ends. *Leptospira *comprehends more than 30 serotypes and 350 serovars that could infect both humans and animals with a diverse degree of damage to their host [5]. These are furthermore grouped into pathologic, intermediate pathologic, and saprophytic groups; the interrogans, kirschneri, and the noguchii are some of the more clinically relevant groups of pathological *Leptospira*. Despite being under-diagnosed by the difficulties of laboratory detection and data collection, especially in endemic territories due to them being mostly resource-poor settings, *Leptospira *is responsible for one million cases worldwide, 60 000 deaths, and a morbidity rate of 14.8 cases per 100,000 people annually, having a significant impact worldwide [5,6]. *Leptospira *disease is endemic in Asia and South America and is mostly unattended in the pacific region like Oceania, the most drastically affected area by leptospirosis according to a systematic review by Guernier et al., 2018 [7]. The prevalence is higher in tropical and subtropical regions with the majority of cases in developing countries; in Ecuador, the public ministry of health reports an incidence of one case per 100,000 inhabitants. A higher incidence is favored by natural disasters that are becoming more frequent, such as floods, hurricanes, and tropical storms [6].

The clinical scenario for leptospirosis varies widely, and its diagnosis requires a high clinical suspicion [8]. In this case, the differential diagnosis had to be centered on diseases associated with an occupational risk of exposure to vector-borne diseases, with fever, elevated liver enzymes, and hyperbilirubinemia as presenting factors. All tropical zoonoses were considered in this differential diagnosis as well as COVID-19 infection due to the high rates of incidence by the time the case happened [9-11]. Another suspected etiology was a bacterial, parasitic, or less frequent fungal hepatic abscess. In western countries, the hepatic abscess etiology is most commonly due to bacterial infection; the incidence of hepatic abscess is 2.3 cases per 100 000 patients [12], although etiology is not well established nor documented in Latin-American countries. In the context of our patient due to the high prevalence of parasitic and mostly amebic infection in Latin America, it was plausible to think of an amebic liver abscess [13,14]. Parasitic infections of the biliary tract of the nematode Ascaris lumbricoides may lead to biliary obstructions and cause acute cholangitis, which could have also fitted with our patient’s clinical scenario [15,16]. Additionally, the patient had no additional risk factors that could lead to microbial dissemination such as recent appendicitis, history of surgery, diverticulitis, colitis, inflammatory bowel disease, or immunosuppression [12,17]. The key factor, in this case, may rely on the epidemiologic exposures due to the patient’s occupation. In Table 2, we present the different entities to consider in the differential diagnosis regarding this case.

**Table 2:** Differential diagnosis regarding fever of unknown origin, elevated liver enzymes, and hyperbilirubinemia Source: Data for this table was extracted from References [8,9,18].

Vector-Borne Diseases
Dengue
Malaria
Chikungunya
Zika virus
Rickettsial diseases
Yellow fever
Leishmania
Q fever
Other Infectious Diseases
SARS-CoV
Hepatic abscess (bacterial, parasitic, or fungal)
Tuberculosis
Viral hepatitis
Influenza
Typhoid fever
Toxoplasmosis
Other
Alcohol hepatitis
Cholangitis
Biliary tract obstruction
Liver metastasis

In our case, the patient had an uncommon presentation of leptospirosis, a zoonotic disease that frequently affects biologists; the most common symptoms of this disease are fever of acute onset usually accompanied by malaise, chills, myalgia, conjunctival suffusion, anorexia, abdominal pain, nausea, and vomiting [19,20]. Clinical presentation is variable (Table 3 presents information extracted from References [21-24]) and nonspecific. Studies found that up to 40% of cases of fever of unknown origin in endemic regions are due to infection by *Leptospira *spp [20]. Our patient only presented with fever, malaise, and abdominal discomfort, which contributed to the delay in his diagnosis.

**Table 3:** Relative prevalence of symptoms among patients diagnosed with leptospirosis Source: Data for this table was extracted from References [21–24].

Symptoms	Average Prevalence (%)	Range (%)
Fever	94.5	87,5–99
Myalgia	75.5	40–100
Headache	82.3	50–98
Chills	82.5	78–87
Anorexia	77.0	46–92
Nausea	53.0	29–77
Vomiting	42.4	18–73
Arthralgia	40.0	23–59
Diarrhea	27.4	11–53
Abdominal pain	36.7	26–51
Backache	51.0	51
Jaundice	46.1	0–100
Conjunctival suffusion	61.2	28–99
Nuchal rigidity	19.5	12–27
Oliguria or anuria	26.0	26
Hepatomegaly	37.6	15–83
Splenomegaly	21.3	17–25
Rash	7.8	0–25

The transmission of leptospirosis occurs through contact of disrupted human external barriers with surfaces contaminated with the spirochete [6]. It is rarely acquired by ingestion or inhalation of contaminated water, and there have been rare cases of leptospirosis being contracted through animal biting or human-to-human [5]. The most common reservoir for these bacteria has been found to be rodents’ renal tubules, although it can be found among other mammals as well, and when their urine is excreted, it contaminates water or soil creating foci where *Leptospira *can be spread by rain into water ponds, sewages, and lakes allowing further transmission of the bacterium. *Leptospira *is capable of surviving in water, and a pH above 7 is favorable to it, which allows it to survive for weeks after the contamination occurred, making cross-contamination with humans easier, given the lack of access to potable water in the tropical and subtropical regions of developing countries [4,6,7]

The pathophysiology of disease caused by these bacteria is through adhesion to macromolecules of the extracellular matrix of endothelial cells, where it uses collagenases and invasive enzymes to reach the bloodstream and disseminates and infects further organs and tissues evading phagocytosis, resulting in cellular apoptosis by damaging the cell membranes [25]. Host dysregulation and the excessive inflammatory response have been linked to life-threatening organ failure scenarios as leptospirosis is constantly triggering inflammatory responses in humans by inducing the production of pro-inflammatory cytokines [26].

The incubation period for *Leptospira *ranges from two to 20 days, with a median of 10 days [10]. It has an acute bacteremia phase and a delayed immune phase, with the former lasting about a week, and is characterized by the presence of leptospires in the bloodstream [10]. The latter is characterized by the production and presence of circulating antibodies in the bloodstream and leptospires in urine [10]. At this time, bacteria migrate from blood vessels into the host organs, and the disease can be further classified as icteric or anicteric: anicteric disease being the most prevalent and less severe manifestation of the disease, occurring in about 90% of cases [10]. The icteric form of the disease is also known as Weil's disease, which happens in about 5%-10% of cases and has a mortality that ranges from 5% to 40% [27]. In our case, the patient was infected during his trip in the jungle, which occurred 21-30 days before his symptom onset, a much larger incubation period than reported. Additionally, he never presented with jaundice, despite having elevated bilirubin. The only symptomatology was fever, malaise, and abdominal discomfort, and, as shown in Table 3, he did not present other common symptoms such as headache, anorexia, myalgia, vomiting, organomegaly, etc. Finally, his presentation had the same characteristics as a previous SARS-CoV-2 infection he had, showcasing the unspecificity of the symptoms he presented and the reason for the delayed diagnosis.

**Figure 2:**
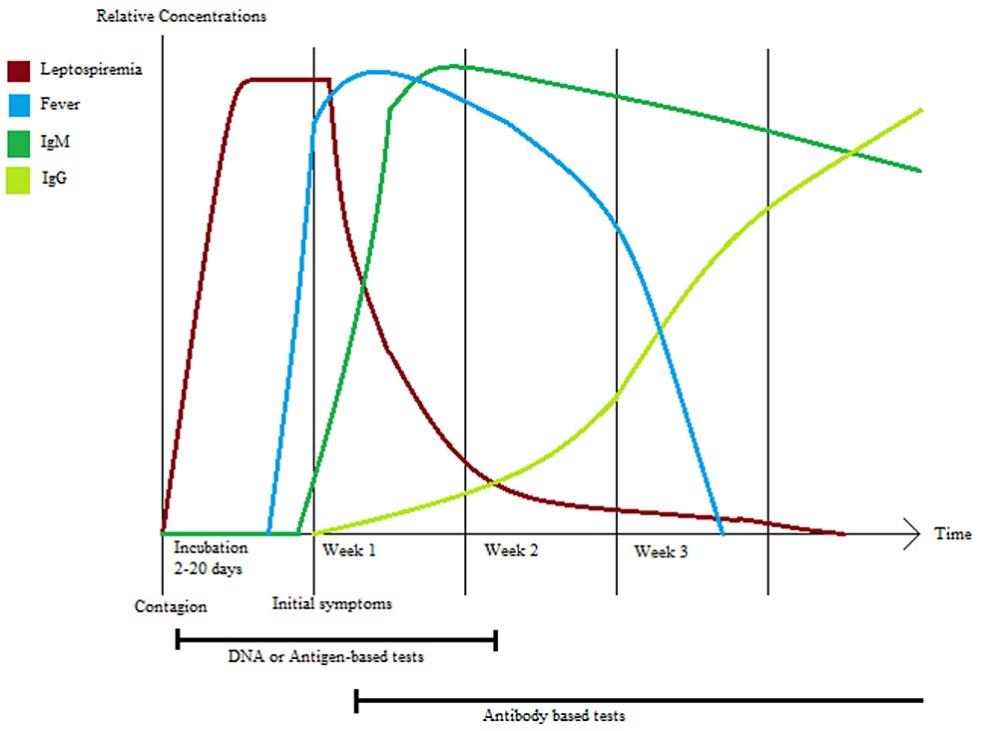
Graphic of the kinetics of leptospiral infection in blood Source: Data for this graph was extracted from Reference [28].

Diagnosing leptospirosis is still a challenge. Isolation and culture-based methods aim to prove the presence of leptospires in a target tissue. A blood sample culture would only be positive if taken during the septicemic phase [10]. However, these methods are time-consuming and labor-intensive and therefore not routinely done. Serology-based tests assess the presence of IgM and IgG antibodies in the blood serum. These tests can be used during the acute phase of illness but require later reconfirmation in endemic areas [10]. A graph showing the antibody kinetics of leptospiral infection is shown in Figure 2. The microscopic agglutination test (MAT) is considered the most sensitive and specific test but requires a highly trained technician and is highly labor-intensive, reasons for which it is falling out of favor [10]. There is also enzyme-linked immunoassay (ELISA) for the detection of *Leptospira*-specific antibodies, which is as sensitive and specific as MAT. This test requires a lower concentration of IgM antibodies than MAT for a positive result. It is less labor-intensive and does not require a highly trained technician, so it is gaining favor as the preferred test for diagnosis [27]. Our patient, despite being assessed since the beginning of symptoms, constantly showed negative test results. Blood culture was taken four days after symptom onset along with the determination of IgM and IgG, a time that, as shown in Figure 2, will normally have leptospiremia showcased by the increase of IgM antibodies in the blood. However, our patient’s case shows that leptospiral infection is difficult to diagnose and that a single negative test result is not sufficient to rule out the disease, even in uncommon cases.

Treatment of leptospirosis continues to be a topic of discussion as the varying clinical presentations make it difficult to reach a consensus. Supportive treatment will vary according to the clinical presentation. There is no clear indication of when to use antibiotics and what antibiotic to use in a leptospirosis disease. Although symptoms range from mild to severe intensity, antibiotic treatment is usually indicated [29-31]. Penicillins are given as the drug of choice and recommended by the World Health Organization, but usually, stronger antibiotics are given in the hospital for broadening coverage as the first line of therapy. Cephalosporins, doxycycline, and chloramphenicol have also been used to treat leptospirosis [5]. Doxycycline and azithromycin are options for a low-severity infection and outpatient treatment [5]. Though there is a wide range of antimicrobial options, none has shown effectiveness regarding dialysis treatment necessity, jaundice, and liver function improvement [29-31]. Thus, preventive measures regarding rodent control, sanitary policies, and food and water regulations have a higher impact on reducing mortality [6]. Doxycycline and penicillin are the most effective chemoprophylaxis therapies used in endemic regions, although there is no significant evidence regarding their effectiveness if the patient is found in an advanced stage of the infection [29]. Vaccines currently under research seem to be promising, but there is no long-lasting immunity. The diversification of serotypes of *Leptospira *and the lack of understanding of its pathogenesis are an issue regarding the creation of a universal vaccine for all subtypes [5].

Patient’s perspective

The patient shared in an interview that there were feelings of stress and anxiety as the disease was not diagnosed early on. While the initial diagnosis was COVID-19 disease, repeated tests discarded this possibility, adding a melancholic and hopelessness component as each result came back negative until the final diagnosis was made 10 days after initial symptom onset. It is important to note that the patient did not require any psychological consultation after the episode, and the feelings reported have subsided.

Additionally, the patient manifested that he understood that COVID-19 disease can be confused as a main etiology/cause of fever of unknown origin, but he wished that his case was managed in a more specific manner.

## Conclusions

Given the wide spectrum of leptospirosis that ranges from asymptomatic to systemic inflammatory response syndrome, leptospirosis may be overlooked as a potential differential diagnosis of fever of unknown origin, especially with the current high prevalence of COVID-19. Furthermore, people who reside in non-endemic areas of leptospirosis may contract this disease by occupational exposure, and without a clear medical history, the diagnosis can be delayed even more. People who do not live in endemic areas of leptospirosis may contract it and have a delayed diagnosis and treatment. Serology may be falsely negative, and without clear exposure history, it is challenging to diagnose without performing repeated testing.

Health-care professionals must take into account the “COVID-19 delusion” for establishing an adequate differential diagnosis so that suspected etiologies that present as fever of unknown origin can be ruled out in a timely manner. Overlooking infectious and endemic etiologies can be a burden to health-care systems and certainly can cause distress, anxiety, and even depression in patients as longer than necessary hospital stays with delayed diagnoses can be taxing to the emotional well-being of our patients.
